# Cobalt Chloride Treatment Used to Ablate the Lateral Line System Also Impairs the Olfactory System in Three Freshwater Fishes

**DOI:** 10.1371/journal.pone.0159521

**Published:** 2016-07-14

**Authors:** Julie M. Butler, Karen E. Field, Karen P. Maruska

**Affiliations:** Department of Biological Sciences, Louisiana State University, 107 Life Sciences Bldg., Baton Rouge, LA, 70803, United States of America; University of Auckland, NEW ZEALAND

## Abstract

Fishes use multimodal signals during both inter- and intra-sexual displays to convey information about their sex, reproductive state, and social status. These complex behavioral displays can include visual, auditory, olfactory, tactile, and hydrodynamic signals, and the relative role of each sensory channel in these complex multi-sensory interactions is a common focus of neuroethology. The mechanosensory lateral line system of fishes detects near-body water movements and is implicated in a variety of behaviors including schooling, rheotaxis, social communication, and prey detection. Cobalt chloride is commonly used to chemically ablate lateral line neuromasts, thereby eliminating water-movement cues to test for mechanosensory-mediated behavioral functions. However, cobalt acts as a nonspecific calcium channel antagonist and could potentially disrupt function of all superficially located sensory receptor cells, including those for chemosensing. Here, we examined whether CoCl_2_ treatment used to ablate the lateral line system also impairs olfaction in three freshwater fishes, the African cichlid fish *Astatotilapia burtoni*, goldfish *Carassius auratus*, and the Mexican blind cavefish *Astyanax mexicanus*. To examine the impact of CoCl_2_ on the activity of peripheral receptors, we quantified DASPEI fluorescence intensity of the olfactory epithelium from fish exposed to control and CoCl_2_ solutions. In addition, we examined brain activation in olfactory processing regions of *A*. *burtoni* immersed in either control or cobalt solutions. All three species exposed to CoCl_2_ had decreased DASPEI staining of the olfactory epithelium, and in *A*. *burtoni*, cobalt treatment caused reduced neural activation in olfactory processing regions of the brain. To our knowledge this is the first empirical evidence demonstrating that the same CoCl_2_ treatment used to ablate the lateral line system also impairs olfactory function. These data have important implications for the use of CoCl_2_ in future research and suggest that previous studies using CoCl_2_ should be reinterpreted in the context of both impaired mechanoreception *and* olfaction.

## Introduction

Fish constantly receive information via multiple sensory channels and the specific role of these different senses is a common focus of fish sensory biology and neuroethology. To examine what role individual senses such as vision, audition, mechanoreception, and chemoreception have in mediating behaviors, a typical approach is to disrupt input from a single sensory system (e.g. eye patches to remove visual cues) and then compare behavioral responses to those from undisrupted intact fish. It is extremely important, therefore, to verify that the methodology used to ablate/disrupt a sensory system has the desired effects on the intended sense, but also that it does not impair other sensory modalities or have toxicity effects.

The mechanosensory lateral line system allows fish to receive information about their surroundings through detection of near-body flow water movements relative to the skin surface [1–4]. This sensory system is composed of neuromasts, which are groups of support cells and hair cells with their hair bundles (single kinocillium and multiple rows of stereovilli) embedded in a gelatinous cupula [3]. Neuromasts are located either inside bony canals in the dermis (canal neuromasts) or on the skin surface of the fish (superficial neuromasts) [5, 6]. Mechanosensory cues are used during schooling [7,8], rheotaxis [7,9–11], prey detection [12–14], predator avoidance [15,16], and social interactions [17–20], and the importance of mechanoreception during these behaviors has been primarily examined by chemically ablating the lateral line system using aminoglycoside antibiotics (mechanotransduction channel blocker) or cobalt chloride (putative calcium channel antagonist [21–23]). While it has become more common for studies using chemical ablation to verify treatment efficacy in recent years, it is still rare for studies to examine potential effects on other sensory systems. Specifically, almost no lateral line studies tested for potential impacts of cobalt chloride on chemosensory function, an important sensory modality for many behaviors in fishes [24–27]. Without these verifications, however, conclusions on the specific role of mechanosensation during context-specific behaviors are called into question.

Across taxa, chemical signals provide crucial information for both survival (food, predators) and reproduction (mates, offspring) [28–36]. Because of their aquatic environment, fishes are constantly exposed to water-soluble compounds that are readily detected by chemosensory systems. The two primary modes of chemical detection in fishes are olfaction (smell) and gustation (taste) [24,37]. The teleost olfactory system consists of an olfactory epithelium that lies beneath the nares on each side of the head [38,39]. As water flows over the epithelium, odorant molecules bind to olfactory receptor neurons that then transmit action potentials along the olfactory nerve to the olfactory bulb [40,41]. From here, signals are transmitted by either the lateral olfactory tract (feeding information) or medial olfactory tract (social and alarm information) to higher brain centers including those involved in feeding, social behavior, and reproductive physiology [40]. Taste buds are generally located on the lips, inside the mouth, on the pharyngeal (gill) arch epithelium, and in some fishes on the external body surface, while solitary chemosensory cells are located on the surface of the head, trunk, and tail of fishes [42–44]. By virtue of their location, all of these chemoreceptive cells in fishes are exposed to the aquatic environment, including any toxins, heavy metals, or other water-soluble compounds that are commonly used to chemically/pharmacologically eliminate the lateral line system, such as CoCl_2_. As a non-specific calcium channel antagonist [21–23], it is possible that CoCl_2_ treatment also impairs superficially located chemosensory cells in addition to neuromasts of the lateral line system.

Disruption of chemosensory communication could have extreme effects on many fish behaviors that are crucial for survival and reproductive success. Taste is used for food detection [44–47], but there is little experimental evidence thus far to suggest a role in social communication. The olfactory system, however, mediates many behaviors including feeding, predator avoidance, migration, and kin recognition, and is used to detect substances released from conspecifics during social interactions such as aggression and courtship [24,48]. Although it is likely that chemosensory information is used by many fishes for survival and social interactions, the relative importance of olfactory and gustatory stimuli in behaviors such as feeding in the dark and reproduction still needs to be evaluated on a species by species basis. Because chemoreception is a vital sense for many fish behaviors, it is extremely important to verify that these systems (taste and smell) remain intact following chemical/pharmacological manipulation of other sensory systems.

The effects of heavy metals (e.g. copper, cobalt) on fish behavior and olfaction have been well studied due to their occurrence as environmental contaminants (reviewed in [49]). Copper has been extensively studied and has known detrimental effects on fish olfaction [50–53]. However, the role of cobalt remains less clear. While some studies have found altered behavior and olfactory function following cobalt exposure [22,54–56], other studies have found that sub-lethal levels of cobalt had little to no impact [57,58]. It is possible that the reason for these varied results is due to species-specific responses to cobalt (evidenced by the need for various treatment protocols: e.g. [14,17,19–21,59]) as well as differences in their exposure protocols. While past studies in fishes have used cobalt concentrations consistent with environmental levels or short-term exposures (<1 hour) during assessment of behavioral and olfactory responses, no studies have tested if the same cobalt chloride treatments used to ablate the lateral line system also impair the olfactory system.

The goal of this study was to examine the effects of CoCl_2_ on the mechanosensory lateral line and olfactory systems in three freshwater fish species: an African cichlid fish (*Astatotilapia burtoni*), goldfish (*Carassius auratus*), and the Mexican blind cavefish (*Astyanax mexicanus*). These species were chosen because they are representatives of the three primary taxa used in current lateral line research (i.e. cichlids, goldfish, cavefish), and are commonly subjected to CoCl_2_ treatment. By evaluating each sensory system peripherally (assessment of vital-dye staining) and centrally (assessment of activation of brain processing regions), a better understanding can be gained of the potential impacts of cobalt chloride on multiple sensory systems.

## Materials and Methods

### Experimental animals

Adult *Astatotilapia burtoni* were laboratory-bred from a wild-caught stock collected from Lake Tanganyika, Africa in the 1970s [60], and were maintained in an environment that mimicked their natural conditions. *A*. *burtoni* were housed in 30L aquaria at 28–30°C on a 12L:12D diurnal cycle and fed cichlid flakes (AquaDine, Healdsburg, CA) once daily and supplemented with brine shrimp. Goldfish and cavefish were bought from local pet suppliers (Petco store #1580, Baton Rouge, LA; geographic coordinates: 30°25’17”N 91°09’12”W; purchased December 2015) and PetSolutions (Beavercreek, OH; www.petsolutions.com; purchased December 2015), respectively. Goldfish were of mixed color morphs. Goldfish and cavefish were acclimated to and housed in 3L buckets containing aerated RO (reverse osmosis) water for ~2 hours before treatment began. RO water, as opposed to salt-supplemented water, was used because goldfish acclimated to and housed in cichlid-system water (i.e. RO water with salt supplementation) during preliminary studies appeared sluggish and some even died. However, goldfish placed in RO water had no adverse effects. All animals were used to examine peripheral impacts of cobalt chloride (CoCl_2_), but only *A*. *burtoni* were used to examine the effects of CoCl_2_ on central processing. All experiments were performed in accordance with the recommendations and guidelines stated in the National Institutes of Health (NIH) Guide for the Care and Use of Laboratory Animals, 2011. The protocol was approved by the Institutional Animal Care and Use Committee (IACUC) at Louisiana State University, Baton Rouge, LA.

At the end of experiments, fish were measured for standard length (SL), weighed for body mass (BM), and gonads were removed and weighed to calculate gonadosomatic index (GSI) as a measure of reproductive status [GSI = (gonad mass/BM)*100]. Animals were of mixed sex (*A*. *burtoni*: 34 males, 19 females; goldfish: all males; cavefish: 17 males, 7 females, 3 immature) and body size (*A*. *burtoni*: SL 51.019±1.397mm, BM 4.340±0.321g; goldfish: SL 36.131±1.170mm, BM 1.772±0.859g; cavefish: SL 29±0.959mm; BM 0.615±0.049g). None of these variables (BM, SL, GSI, sex) impacted fluorescence intensity of the olfactory epithelium or efficacy of CoCl_2_ treatment (Pearson correlation: P>0.05 for all factors in all species), and did not differ among groups (one-way ANOVA, P>0.05 for all).

### Cobalt chloride treatment

*A*. *burtoni* were assigned to one of four groups: two CoCl_2_ treatment groups to assess the impact of low (0.1mM CoCl_2_ for 3 hours) and high (2mM CoCl_2_ for 3 hours) doses and two control groups (one with and one without a calcium-chelator) to examine the impact of low-calcium water. The two control groups together are referred to as ‘controls’ or separately as ‘untreated-control’ (no calcium chelator) and ‘low-calcium’ (calcium chelator present). Animals assigned to the untreated-control group were immersed in normal cichlid-system water (RO water supplemented with Lake Tanganyika buffer to pH8.0, and salt to 300–500μS/cm; total calcium: 250–500μM) for 3 hours and was primarily included as a reference for DASPEI fluorescence quantifications of the olfactory epithelium (see below). Fish assigned to the other control group, low-calcium, were immersed in cichlid-system water with 1mM EGTA (ethylene glycol tetraacetic acid; calcium-chelating agent used to lower water calcium levels as done previously [17–19]) for 3 hours. This low-calcium control group was needed because the CoCl_2_ treatment solutions are prepared in low-calcium water. CoCl_2_-treated fish were immersed in cichlid-system water containing 1mM EGTA and 0.1mM CoCl_2_ (low) or 2mM CoCl_2_ (high) for 3 hours. Sodium hydroxide (NaOH) was used to restore pH to 7.6–8.0 for all solutions. Total calcium concentrations were measured using flame emission spectroscopy, and the amount of free calcium was determined using freely available EGTA-Ca^2+^ calculators (e.g. http://randombio.com/egta.html; http://maxchelator.stanford.edu/CaEGTA-TS.htm). EGTA bound >99% of the total calcium in the cichlid-system water indicating that all EGTA-containing solutions (i.e. low-calcium, low-0.1mM CoCl_2_, and high-2mM CoCl_2_) are effectively calcium-free. Individual fish were placed in glass beakers containing 500mL of the treatment solution and an air stone. In most freshwater fishes, immersion in 0.1mM CoCl_2_ for ~24 hours is sufficient to functionally ablate the lateral line system [52], but there is species-specific variation in treatment efficacy. In *A*. *burtoni*, immersion in 0.1mM CoCl_2_ for 24 hours results in minimal ablation of the lateral line neuromasts, whereas an increased dose of 2mM CoCl_2_ for 3 hours completely ablates cranial canal and superficial neuromasts [17]. In the peacock cichlid, however, treatment with 0.1mM CoCl_2_ for 3 hours ablates the lateral line system [14,61]. Because of this, both low (0.1mM for 3 hours) and high (2mM for 3 hours) CoCl_2_ treatments were tested. Controls for treatment efficacy and toxicity were previously reported for treatment with 0.1mM CoCl_2_ and 2mM CoCl_2_ in *A*. *burtoni* [17].

Goldfish and cavefish were assigned to one of two groups: control or 0.1mM CoCl_2_ treatment. Groups of 6–8 fish were immersed in 1 L of normal RO water (control) or 0.1mM CoCl_2_ prepared in RO water for 24 hours (CoCl_2_-treated). This dose of CoCl_2_ was chosen because it was shown previously to ablate the lateral line system of goldfish and cavefish, while a higher dose (e.g. 2mM) is lethal for both [21,55,59,62]. The lack of salt in the RO water can cause some ionic imbalances for the fish, but since both the control and 0.1mM CoCl_2_ groups were in RO water, this did not affect interpretation of the data. In addition, a low-calcium control was not used for goldfish or cavefish because RO water already contained <10μm free calcium.

Individuals of all fish species ate normally immediately after treatment, had no excess mucus production, and showed normal behaviors, swimming patterns, and coloration. In addition, no fish died as a result of any of the CoCl_2_ treatments. Several CoCl_2_-treated *A*. *burtoni*, goldfish, and cavefish were allowed to live in normal cichlid-system (*A*. *burtoni*) or RO (goldfish and cavefish) water for several weeks after treatment and there were no delayed toxicity effects in any of these individuals.

### DASPEI staining and imaging

To visualize the impact of the CoCl_2_ treatment on the olfactory epithelium, fish were stained with the fluorescent vital dye, DASPEI (2-[4-(dimethylamino)styryl-*N*-ethylpyridinium iodine; Invitrogen Molecular Probes). DASPEI is a mitochondrial stain taken up by metabolically active cells, can be used as a measure of living or viable cells within a tissue, and its fluorescence intensity is qualitatively correlated with mitochondria activity and number [63]. Other styryl dyes, such as FM1-43, have been show to enter sensory cells via mechanotransduction and ion channels [64], and their fluorescence intensity can be reflective of the amount of dye taken into and retained by the cells. Although we are unsure if the measured fluorescence intensity is representative of the mitochondrial activity or intake through ion channels, the observed fluorescence acts as a measure of overall cell activity and will only stain alive, functioning cells. Fish were immersed in 0.008% DASPEI in RO water for 20 minutes to visualize lateral line neuromasts and cells of the olfactory epithelium. Following staining and tissue collection (see below), all animals were imaged using an eGFP filter set (excitation filter 485/20; 525 LP filter) on a stereo dissection microscope (SteREO Lumar V.12, Zeiss) and images were acquired with Zen Pro software. *A*. *burtoni* were stained in groups that always included at least one untreated-control animal to account for any differences in batches of DASPEI solutions and replacement of the fluorescent light source half way through the *A*. *burtoni* data collection, but cavefish and goldfish were all stained at one time in the same DASPEI solution to reduce staining variability.

The olfactory epithelia of DASPEI-stained fish were exposed by gently removing the overlaying tissue around the nares. Extended depth of field imaging was used to obtain images of the entire olfactory epithelium. Images were taken at the same magnification and exposure for all animals that were stained together. All animals were left in a dark container prior to imaging, and all images were taken within 5 minutes of exposing fish to light to avoid photobleaching. Fish were imaged in random order and imaging order had no impact on fluorescence intensity (Pearson correlation: *A*. *burtoni*: R = 0.078, P = 0.599; goldfish: R = -0.075, P = 0.700; cavefish: R = 0.145, P = 0.469).

To investigate if olfactory abilities recover after CoCl_2_ treatment, we examined all fish species immediately following treatment and 18 hours later. Some studies using CoCl_2_ to chemically ablate the lateral line system, especially those using cichlids, allow the fish to recover after treatment (from 2 [14,61] to 18 hours [17]). To better mimic these studies, we allowed fish to recover for 18 hours post-treatment, as done in [17]. Further, this allowed us to examine if the impact of CoCl_2_ on the olfactory system would be alleviated after recovery or if impairment persisted after treatment, which to our knowledge, has never been tested. Fish assigned to the recovery groups were placed in either normal cichlid-system (*A*. *burtoni*; [free Ca^2+^] = 250–500μM) or RO (goldfish and cavefish; [free Ca^2+^] <10μM) water for ~18 hours after which they were quickly netted from their tank and handled as described above for DASPEI staining and imaging.

**Fig 1:**
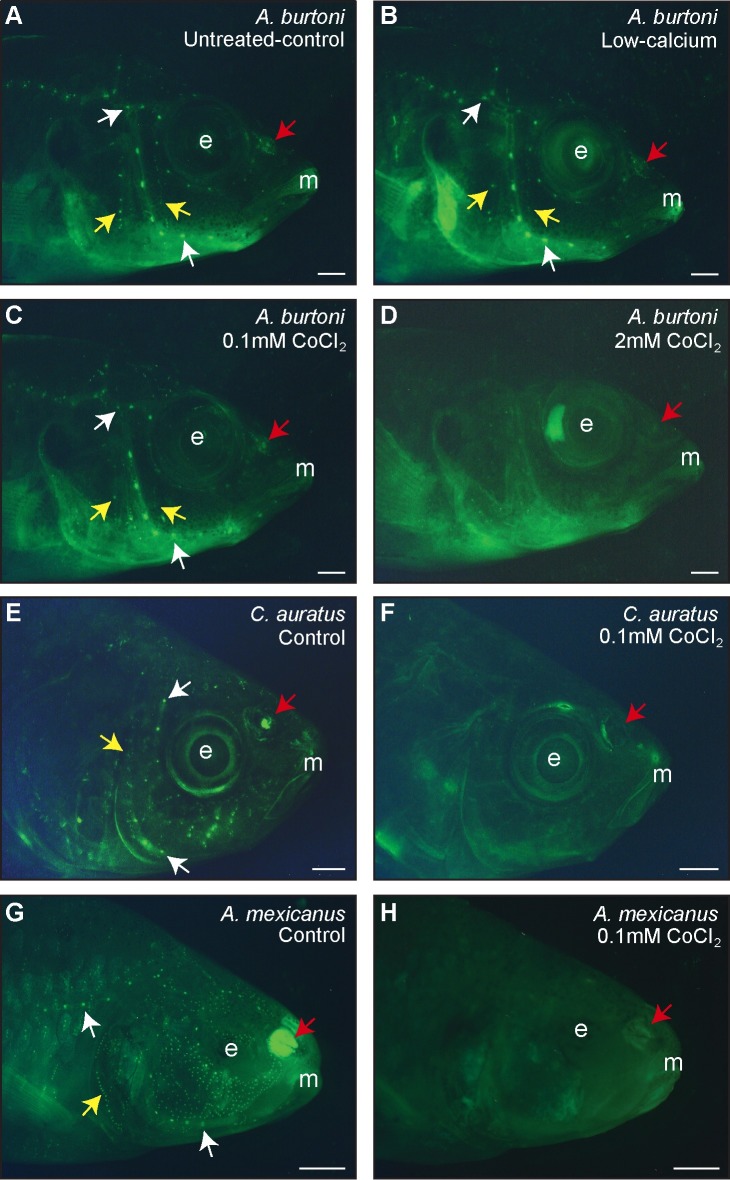
DASPEI staining verified ablation of the lateral line neuromasts and revealed impacts on the olfactory epithelium in three freshwater fish species. Representative photomicrographs of: the cichlid *A*. *burtoni* immersed in untreated-control (**A**), low-calcium (**B**), 0.1mM CoCl_2_ (**C**), and 2mM CoCl_2_ (**D**) solutions; goldfish *C*. *auratus* treated with control (**E**) and 0.1mM CoCl_2_ (**F**) solutions; and cavefish *A*. *mexicanus* immersed in control (**G**) and 0.1mM CoCl_2_ (**H**) solutions. A-D were taken 18-hours post-treatment and E-H were taken immediately after treatment. Small green dots are DASPEI-labeled neuromasts and representative superficial and canal neuromasts are indicated with yellow and white arrows, respectively. Red arrows indicate the olfactory epithelium, which is DASPEI-stained in controls but not in cobalt-treated fish. Scale bars represent 2 mm. *Abbreviations*: E, eye/eye socket; M, mouth.

**Fig 2:**
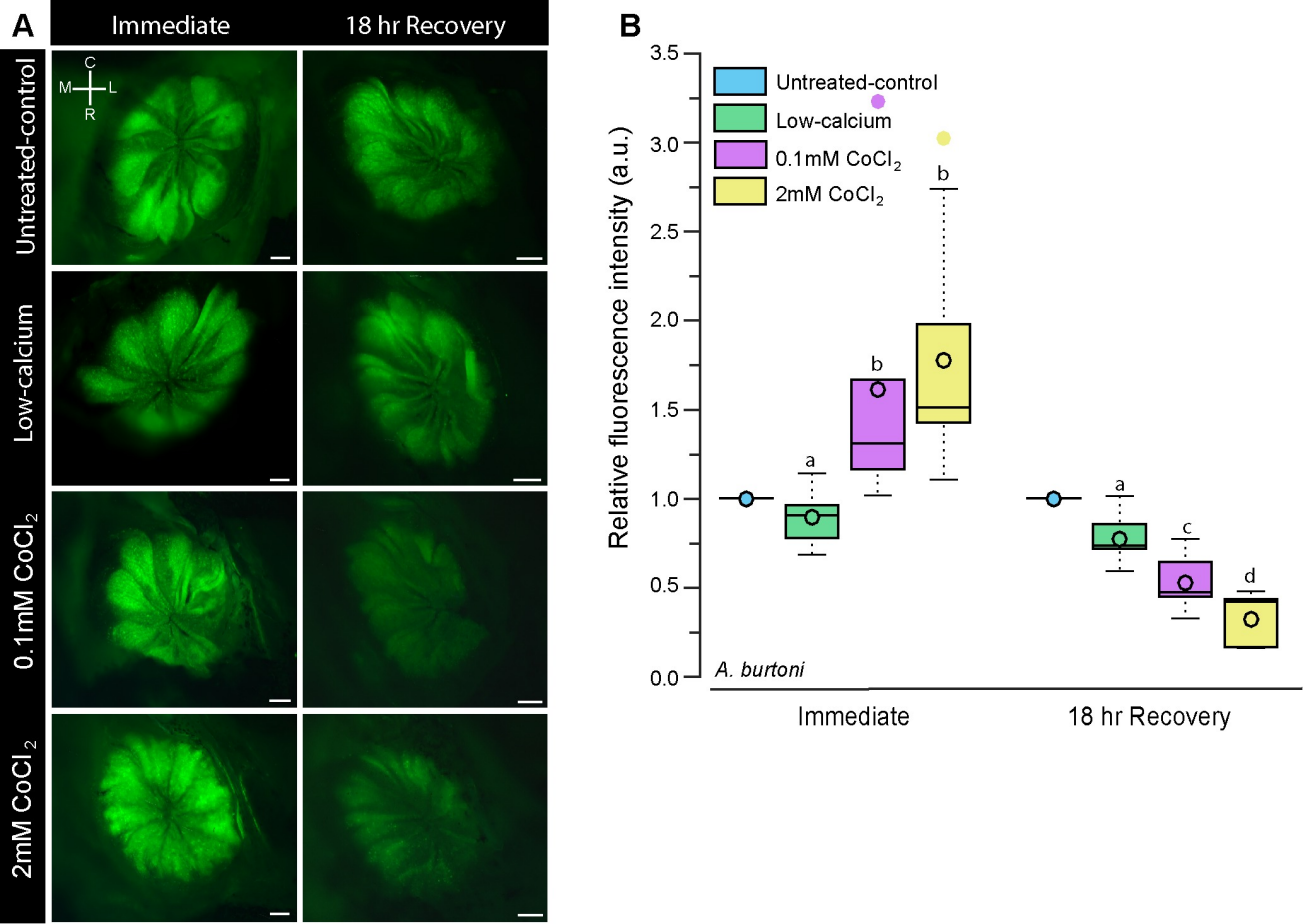
Cobalt chloride treatment reduces DASPEI staining in the olfactory epithelium of the cichlid *A*. *burtoni*. **A**) Representative photomicrographs of DASPEI-stained olfactory epithelia from *A*. *burtoni* immersed in untreated-control water, low-calcium water, low-0.1mM CoCl_2_, and high-2mM CoCl_2_ immediately after treatment and 18-hours post-treatment. **B**) Quantification of DASPEI fluorescence intensity of the olfactory epithelium. Fluorescence intensity was normalized to untreated-controls such that a value = 1 indicates the same fluorescence, >1 indicates greater fluorescence, and <1 indicates reduced fluorescence relative to the untreated-controls. Different letters indicate statistical significance at P<0.05. Tukey’s box plots were used to represent data (N = 6 fish per group): data median is represented by a line and data mean by an open circle, the box extends to the furthest data points within the 25th and 75th percentile, and whiskers extend to the furthest data points not considered outliers. Absence of whiskers indicates absence of data points outside of the 25th/75th percentile. Outliers are represented by closed circles. Scale bars in A represent 200μm. *Abbreviations*: C: caudal, L: lateral, M: medial, R: rostral.

**Fig 3:**
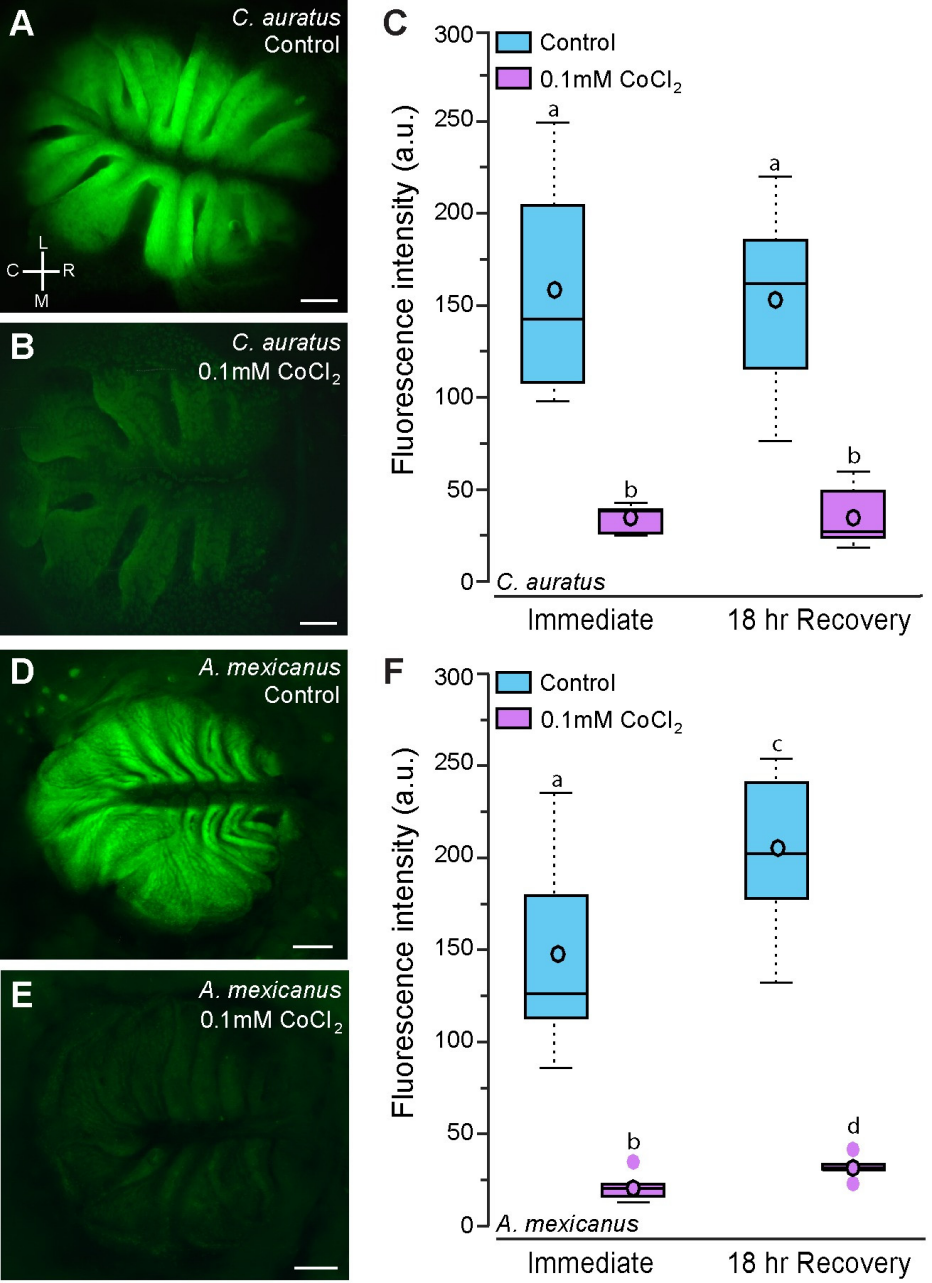
Goldfish and cavefish treated with CoCl_2_ have reduced DASPEI staining of the olfactory epithelium. Representative photomicrographs of the olfactory epithelium of control (**A**) and CoCl_2_-treated (**B**) goldfish, *C*. *auratus*. **C**) Quantification of DASPEI fluorescence intensity of the olfactory epithelium of control and CoCl_2_-treated *C*. *auratus* (N = 7–8 fish per group). Representative photographs of the olfactory epithelium of control (**D**) and CoCl_2_-treated (**E**) Mexican blind cavefish, *A*. *mexicanus*. **F**) Quantification of DASPEI fluorescence intensity of the olfactory epithelium of control and CoCl_2_-treated *A*. *mexicanus* (N = 6–7 fish per group). Different letters indicate statistical significance at P<0.05. Scale bars in A, B, D, and E represent 200μm. See Fig 2 for box plot descriptions. Abbreviations: C: caudal, L: lateral, M: medial, R: rostral. Orientation in A also applies to images in B, C, and D.

Following alignment of extended depth of field images on the stereomicroscope, artifacts (small white dots) appeared in all aligned images. Artifacts were consistent across images (each image had 2–3) and were included in fluorescence intensity quantifications. For visualization purposes in Figs 1–3, artifacts were removed in Photoshop CS6. All images had the same color balance and contrast settings applied.

### Fluorescence intensity quantification

Once EDF stacked images of the olfactory epithelium were acquired, a region of interest (ROI) was outlined as the circumference of the olfactory epithelium. Zen Pro software was then used to calculate fluorescence intensity (FI) for the region of interest. Background fluorescence was accounted for by subtracting the FI of the background from the FI of the region of interest. This normalizes fluorescence values by accounting for individual variability in DASPEI staining. To eliminate inter-group variability in *A*. *burtoni*, each value was divided by the FI of the untreated-control animal (relative FI = FI of subject divided by FI of untreated-control) to give a relative FI for each fish such that a value >1 indicates greater fluorescence and a value <1 signifies reduced fluorescence compared to untreated-control animals. Consequently, all untreated-control animals had a relative fluorescence value of 1. The low-calcium fish could not serve as the reference animal as this would have excluded them from statistical analyses. Imaging and quantification was done unilaterally and blind to the animal’s group. ROI area had no impact on FI measurements (Pearson correlation: *A*. *burtoni*: R = -0.097, P = 0.511; goldfish: R = 0.145, P = 0.452; cavefish: R = 0.372, P = 0.056).

Since the goal of this study was to measure effects of CoCl_2_ on the olfactory system, we only qualitatively examined DASPEI staining in the lateral line neuromasts. We were unable to quantify neuromast fluorescence intensity because handling during brain collection removed superficial neuromasts and the tissue above canal neuromasts was difficult to image through, which produced inconsistent fluorescence intensities. Instead, we refer readers to previous research documenting the impact of similar cobalt chloride concentrations on lateral line function in all three species [17,18,55,59,65].

### Tissue collection

DASPEI fluorescence intensity provides information on the impact of CoCl_2_ treatment on peripheral tissues (i.e. olfactory epithelium) but provides no information on central processing. To test whether CoCl_2_ treatment also had central effects (i.e. decreased processing of chemosensory cues) in *A*. *burtoni* we used *in situ* hybridization for the immediate early gene *cfos* as a proxy for neural activation (see details below) in the same animals used for DASPEI imaging. Following DASPEI staining, all fish were quickly netted from the staining solution, anesthetized in ice cold fish water, measured as described above, and killed by rapid cervical transection. Heads from goldfish and cavefish were immediately placed in dark containers of RO water until imaging. For *A*. *burtoni*, the brains were removed and the heads placed in a dark container of RO water until imaging. Dissections took less than 5 minutes per animal and order of dissection was randomized and had no impact on DASPEI or *cfos* staining (P>0.05 for all groups). *A*. *burtoni* brains were fixed overnight at 4°C in 4% paraformaldehyde (PFA) in 1x phosphate-buffered saline (1xPBS), rinsed for 24 hours in 1xPBS, and cryoprotected overnight in 30% sucrose in 1xPBS. Brains were then embedded in OCT media (TissueTek), sectioned in the transverse plane on a cryostat at 20μm, and collected onto 2 alternate sets of charged slides (VWR superfrost plus). Slides were dried flat at room temperature for 2 days prior to storage at -80°C.

### Preparation of DIG-labeled riboprobe and *in situ* hybridization

The immediate early gene *cfos* can be used as a marker of neural activation and has been successfully used for this purpose in *A*. *burtoni* [18]. *Cfos* is a transcription factor that is rapidly induced following a variety of stimuli, and several studies show that genomic responses measured via immediate early gene expression correlates with neural activity (i.e. action potentials) measured using electrophysiological techniques (e.g. [66–68]). To visualize *cfos* mRNA in the brain, we used chromogenic *in situ* hybridization with a riboprobe specific to the *A*. *burtoni cfos* mRNA sequence as previously described [18]. Briefly, commercially synthesized *A*. *burtoni cfos* primers (Life Technologies; forward primer: 5’-agagaactgatcgggagcagcgct-3’; reverse primer: 5’-caggttgggatatcattctgcagg-3’) were used to generate a probe template by PCR amplification of brain cDNA, and a transcription reaction was used to incorporate DIG-labeled nucleotides into the purified PCR template before probe purification. The probe was diluted 1:5 in hybridization buffer and stored at -20°C until use. Probe specificity was previously demonstrated [18].

*In situ* hybridization was performed to visualize and quantify activation differences in chemosensory processing regions of the brain between control and CoCl_2_-treated *A*. *burtoni* as previously described [18,69]. Briefly, slides of cryosectioned brains were prepared by sequential washes at room temperature of 1xPBS, 4% PFA, 1xPBS, proteinase K solution, 1xPBS, 4% PFA, 1xPBS, milliQ water, 0.25% pure acetic anhydride in 0.1M triethanolamine-HCl pH 8.0, and 1xPBS. Slides were then incubated in pre-warmed hybridization buffer at 60°C for 3 hours prior to hybridization with *cfos* riboprobe in a humidified chamber at 60°C for 12–16 hours. After stringency washes were performed at 60°C, slides were washed at room temperature in maleate buffer with tween-20 (MABT), blocked in MABT with 2% bovine serum albumin for 3 hours at room temperature, and incubated in alkaline-phosphatase-conjugated anti-DIG Fab fragments overnight at 4°C in a humidified chamber. Slides were then rinsed in MABT at room temperature, incubated in alkaline phosphatase buffer, and developed with nitro-blue tetrazolium / 5-bromo-4-chloro-3’-indolyphosphate (NBT/BCIP) substrate at 37°C in the dark for 2.5hrs. Slides were then rinsed in 1xPBS, fixed in 4% PFA, washed with 1xPBS and coverslipped with aquamount media.

### Quantification of brain activation

To quantify differences in *cfos* staining, slides were visualized on a Nikon Eclipse Ni microscope and photographs were taken with a color digital camera controlled by Nikon Elements software. Brightfield and phase contrast were used to visualize neuroanatomical markers and brain nuclei in relation to DIG-labeled cells. A cresyl violet-stained *A*. *burtoni* reference brain, *A*. *burtoni* brain atlas, and other relevant papers [18,70–72] were used to identify candidate regions. Stereotactical and neuroanatomical markers were used to designate the beginning and end of each quantified region to ensure consistency across animals.

For *cfos* quantification in the internal cellular layer of the olfactory bulbs (ICL), we used ImageJ to determine the percent area of the ICL covered by *cfos*-stained cells (protocol modified from the “Quantifying Stained Liver Tissue” protocol available on the ImageJ website, imagej.nih.gov). This method, as opposed to optical density, accounts for the number of stained cells, not the intensity of the staining, which can vary between *in situ*s. All images were taken with the same magnification, exposure, and white balance settings on the highest resolution. Images were uploaded into ImageJ and split into their RGB components (3 images). Threshold was lowered until it reached the edges of the cells, producing a value equal to the percent of the ICL covered by staining. This was done for each of the RGB components and a relative staining value was calculated by averaging the percent of the ICL area stained in the red and green channels. The blue channel was not used because *in situ* hybridization occasionally produced a light blue background which impaired proper quantification in the blue channel. We analyzed the RGB components instead of single greyscale image because background levels affected results obtained from greyscale images. Greyscale images with high background required higher threshold values to distinguish cell borders which resulted in skewed percent-stained values.

For all other brain nuclei, cell density was calculated as previously described [18]. Images were taken at the highest magnification that encompassed the entire area of interest. Nuclei borders were first outlined and then 50μm x 50μm gridlines were applied to each image. The number of *cfos*-stained cells in five randomly selected boxes per section was quantified, and cell density was calculated by dividing the number of cells within the boxes by the area of the boxes. Four consecutive sections were quantified for each region and averaged together for a cell density value of that region in a particular animal. We quantified *cfos* expression in the posterior part of the dorsal telencephalon (Dp) and the rostral portion of the dorsal part of the lateral zone of the dorsal telencephalon (Dl-dr). The Dp receives direct input from the olfactory bulb, is the putative homolog of the mammalian piriform cortex, and Dp neural activation is dependent on olfactory input [73]. *Cfos* staining in the Dl-dr was measured as a control because it is not known to be directly involved in olfactory processing and is located in close proximity to the olfactory bulbs.

### Statistics

See supporting information files for raw data for each species (S1 Dataset). One-way ANOVAs were used to examine differences in standard length, body mass, gonadosomatic index, and other characteristics among groups. Two-way ANOVAs were used to examine the impact of treatment (control vs cobalt) and timing (immediate vs recovery) in each species. Because the untreated-control *A*. *burtoni* data were used to normalize the DASPEI fluorescence data, they are excluded from analysis on the olfactory epithelium, but are included in all other analyses. Pearson correlations were used to examine if any variable (e.g. imaging order, fish size) impacted DASPEI fluorescence intensity of the olfactory epithelium. In addition, Pearson correlations were used to test for relationships between DASPEI staining in the olfactory epithelium of *A*. *burtoni* and central neural activation measured via *cfos-*stained cell density. In cases where assumptions of normality or equal variance were not met, data were transformed prior to analysis.

## Results

### CoCl_2_ treatment efficacy on the lateral line system

Low-calcium water had no effect on DASPEI staining of the lateral line system in *A*. *burtoni* (Fig 1A and 1B). The low-0.1mM CoCl_2_ treatment had minimal effects on *A*. *burtoni* lateral line neuromasts despite its effectiveness at ablating the peacock cichlid lateral line system [14,62]; whereas the high-2mM CoCl_2_ treatment ablated >90% of cranial canal and superficial neuromasts (Fig 1C and 1D). Extending the treatment time to 24 hours instead of 3 hours, as used here, had no increased effect on neuromast ablation, so only the 3-hour exposure was used. Treatment with 0.1mM CoCl_2_ for 24 hours completely ablated superficial and canal neuromasts of both goldfish and cavefish (Fig 1E–1H).

### DASPEI staining of olfactory epithelium

To examine the impact of cobalt chloride treatment on cells of the olfactory epithelium (OE), we quantified fluorescence intensity of DASPEI-stained olfactory epithelium in CoCl_2_-treated and control animals immediately following treatment and 18-hours post-treatment. In *A*. *burtoni*, fluorescence intensity was relative to an untreated-control animal for each batch of DASPEI staining, meaning that all untreated-control animals have a relative fluorescence value of 1. A value greater than 1 signifies higher fluorescence while a value less than 1 indicates reduced fluorescence relative to the untreated-control. Immediately after treatment, low-calcium-treated *A*. *burtoni* had similar fluorescence intensity (0.895±0.064; mean±s.e.m.) to the untreated-control, but fish treated with CoCl_2_ had increased DASPEI fluorescence of the olfactory epithelium (low: 1.493±0.305; high: 1.848±0.240; Fig 2). Although CoCl_2_ treatment caused an initial increase in DASPEI fluorescence relative to controls, after an 18-hour recovery the relative DASPEI intensity was decreased in a dose-dependent fashion (low: 0.553±0.065; high: 0.323±0.056; (2 way ANOVA: treatment: F = 0.303, P = 0.832; timing: F = 41.822, P<0.001; treatment x timing: F = 10.952, P<0.001).

Goldfish and cavefish treated with 0.1mM cobalt chloride for 24 hours had decreased fluorescence of the olfactory epithelium both immediately after treatment and 18-hours post-treatment compared to controls (Fig 3; goldfish, treatment: F = 64.461, P<0.001; timing: F = 0.0462, P = 0.832; cavefish, treatment: F = 108.242, P<0.001; timing: F = 5.524, P = 0.028, treatment x timing: F = 2.499, P = 0.128).

### Neural activation is reduced in chemosensory processing regions after CoCl_2_ treatment in *A*. *burtoni*

**Fig 4:**
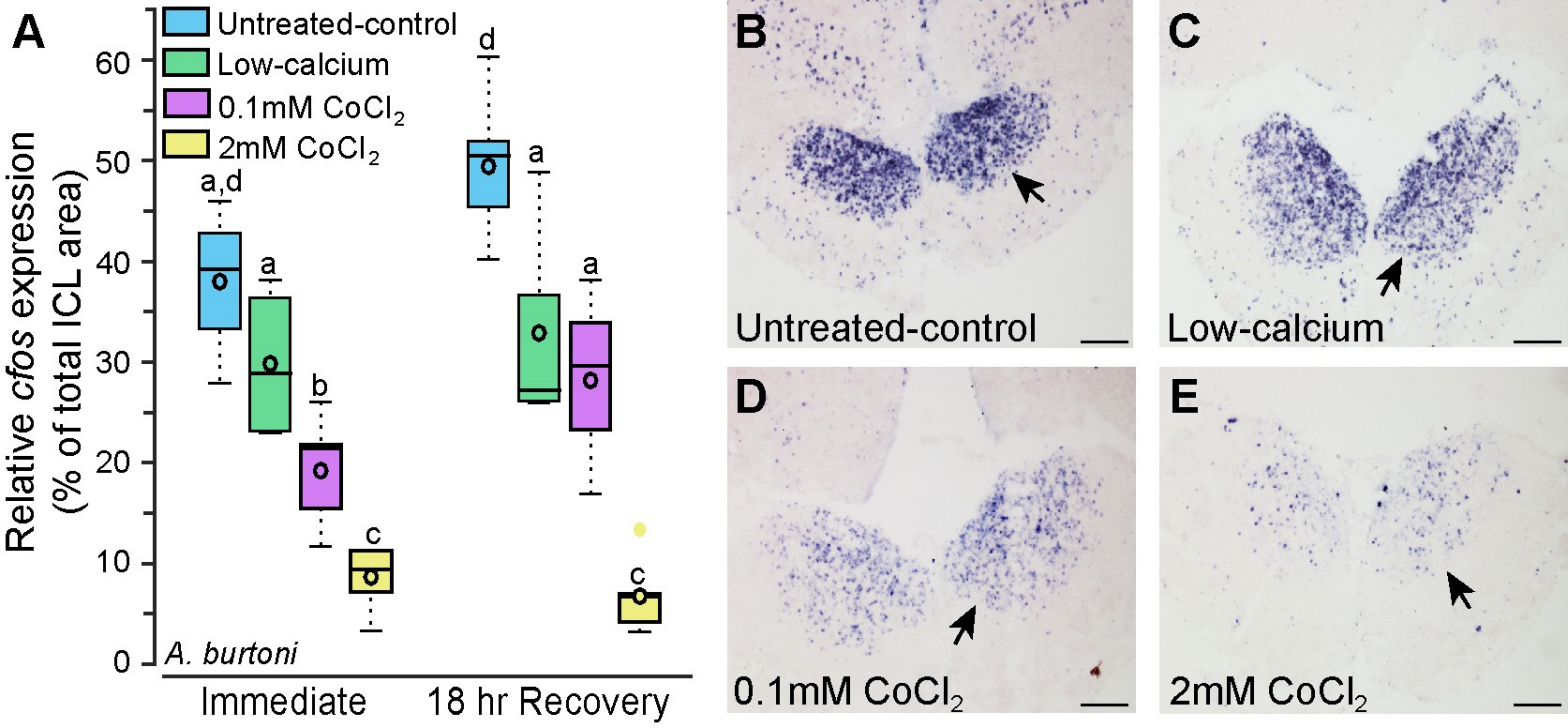
Cobalt chloride-treated *Astatotilapia burtoni* have reduced *cfos* staining in the olfactory bulb. **A**) Animals treated with cobalt chloride have reduced *cfos* staining in the internal cellular layer of the olfactory bulb compared to untreated-control and low-calcium fish (N = 4–6 fish for each group). Photomicrographs of *cfos* staining (purple label) in the olfactory bulbs of representative untreated-control (**B**), low-calcium (**C**), low-0.1mM CoCl_2_-treated (**D**), and high-2mM CoCl_2_-treated (**E**) fish. Arrows indicate internal cellular layer (ICL) of the olfactory bulb. Different letters represent statistical significance at P<0.05. Scale bars in B-E represent 100μm. See Fig 2 legend for box plot descriptions.

We collected brains from *A*. *burtoni* immersed in untreated-control, low-calcium, low cobalt (0.1mM for 3 hours), and high cobalt (2mM for 3 hours) solutions and stained them for the immediate early gene *cfos* as a proxy for neural activation. *Cfos*-stained cell density can be used as a measure of how active that brain region is such that greater numbers of *cfos*-stained cells indicates higher overall activation within a brain region. CoCl_2_-treated animals had reduced *cfos* staining (i.e. activation) in the internal cellular layer of the olfactory bulbs immediately following treatment compared to untreated-control and low-calcium animals, suggesting that olfaction was impaired immediately following CoCl_2_ treatment despite the increased DASPEI staining (Fig 4). *Cfos*-staining reduction was concentration-dependent, such that fish treated with high-2mM CoCl_2_ had fewer *cfos*-stained cells than fish treated with low-0.1mM CoCl_2_ (treatment: F = 40.944, P<0.001; timing: F = 7.705, P = 0.009, treatment x timing: F = 2.306, P = 0.095). Interestingly, 18-hours post-treatment, *A*. *burtoni* treated with low-0.1mM CoCl_2_ had similar *cfos* staining to the low-calcium animals, but both were lower than the untreated-control animals, indicating that both had reduced activation. Fish treated with high-2mM CoCl_2_ still had drastically reduced *cfos* staining at 18-hours post-treatment indicating that this higher dose had prolonged impacts on olfactory functions.

**Fig 5:**
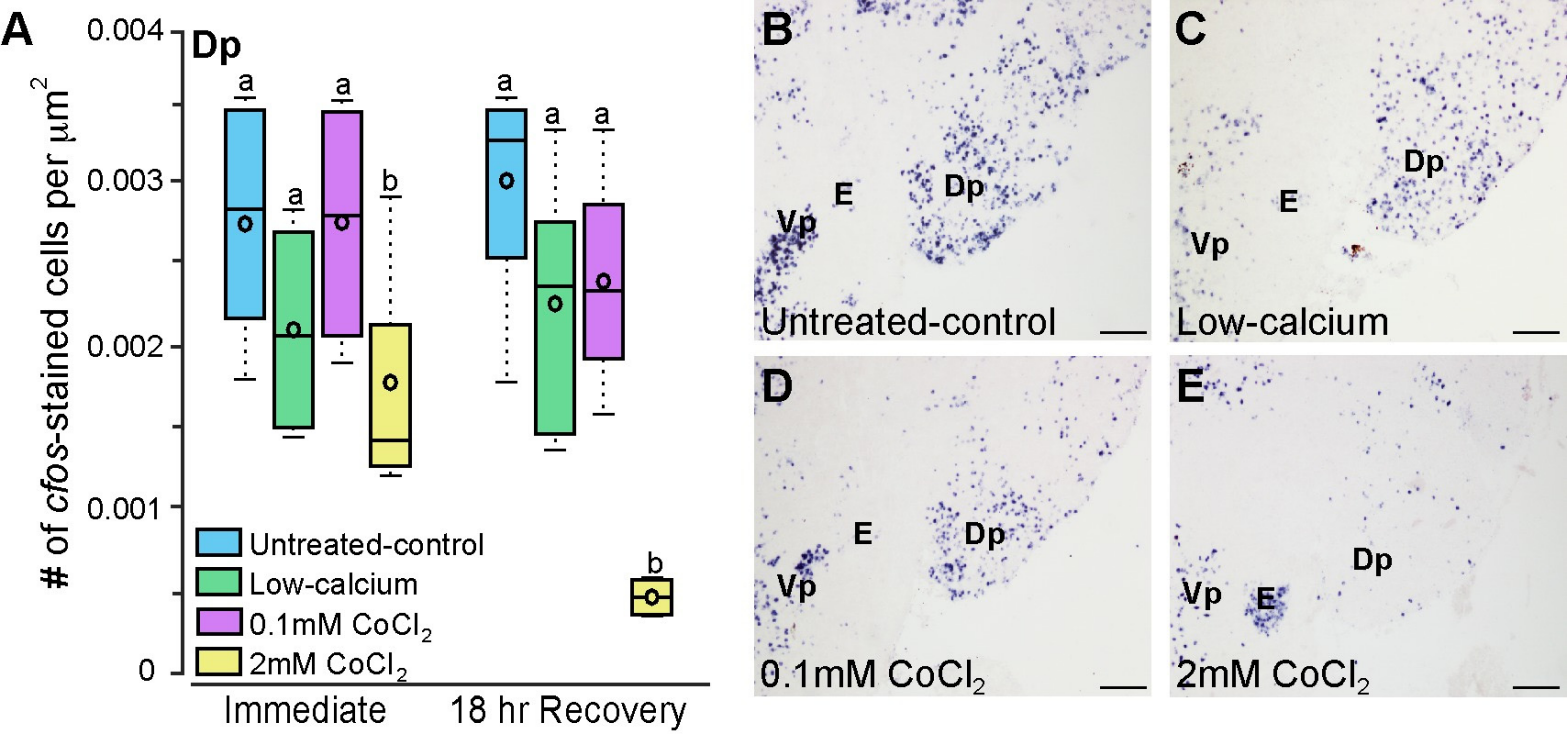
Cobalt chloride-treated *Astatotilapia burtoni* have lower densities of *cfos*-stained cells in the posterior part of the dorsal telencephalon (Dp) at 18-hours post-treatment. **A**) *A*. *burtoni* treated with high-2mM CoCl_2_ have fewer *cfos*-stained cells in the Dp 18-hours post-treatment but not immediately following treatment (N = 4–6 fish for each group). Photomicrographs of *cfos* staining in the Dp of untreated-control (**B**), low-calcium (**C**), low-0.1mM CoCl_2_-treated (**D**), and high-2mM CoCl_2_-treated (**E**) fish allowed to recover for 18 hours. Different letters represent statistical significance at P<0.05. Scale bars in B-E represent 100m. See Fig 2 legend for box plot descriptions. *Abbreviations*: E, entopeduncular nucleus; Dp, posterior part of the dorsal telencephalon; Vp, postcommissural nucleus of the ventral telencephalon.

Olfactory receptor neurons in the olfactory epithelium project to the olfactory bulb where they synapse with mitral [73,74] and tuft cells [75]. The olfactory bulb primarily projects to the posterior part of the dorsal telencephalon (Dp). While there was quite a bit of variability in *cfos* staining in the Dp, fish treated with high-2mM CoCl_2_ had fewer *cfos*-stained cells compared to untreated-control, low-calcium, and low-0.1mM CoCl_2_ fish (Fig 5; treatment: F = 9.683, P = 0<0.001, timing: F = 1.323, P = 0.259, treatment x timing: F = 1.782, P = 0.172). This effect is most evident in the recovery animals, where the high-2mM treated animals have only a fraction of the brain activation of the other groups. This indicates that in addition to the initial synapse in the olfactory bulbs, cobalt chloride treatment affects upstream olfactory processing within the telencephalon.

**Fig 6:**
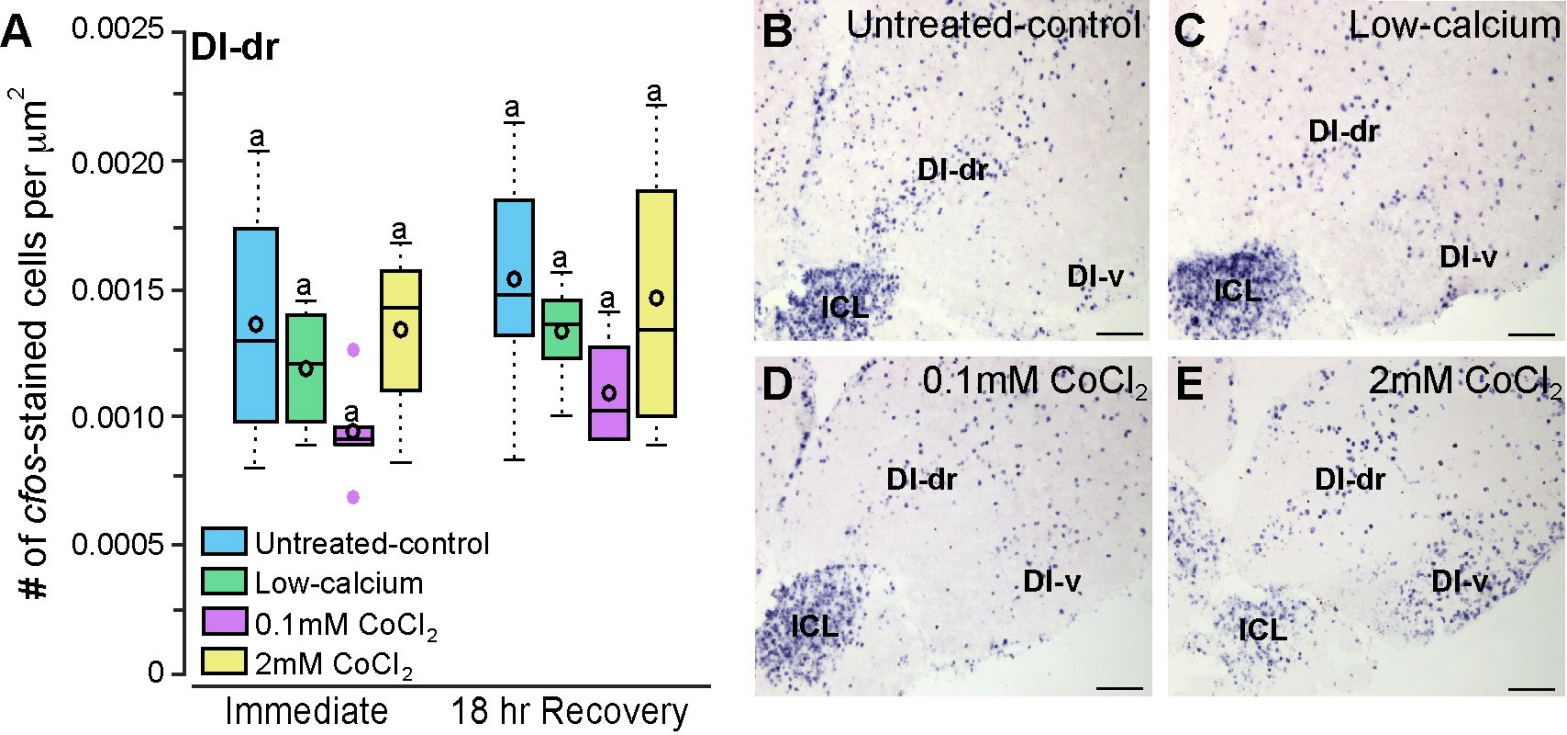
CoCl_2_-treated *Astatotilapia burtoni* had similar *cfos* staining in a control brain region that is not known to process olfactory information. **A**) *A*. *burtoni* immersed in untreated-control, low-calcium, low-0.1mM CoCl_2_, and high-2mM CoCl_2_ solutions had similar numbers of *cfos*-stained cells in the Dl-dr indicating that CoCl_2_ treatment did not affect overall brain activation levels (N = 4–6 fish for each group). Photomicrographs of *cfos* staining in the Dl-dr of untreated-control (**B**), low-calcium (**C**), low-0.1mM CoCl_2_-treated (**D**), and high-2mM CoCl_2_-treated (**E**) fish allowed to recover for 18-hours Same letters represent no statistical significance at P<0.05. Scale bars in B-E represent 10000B0035m. See Fig 2 legend for box plot descriptions. *Abbreviations*: Dl-dr, rostral portion of the dorsal part of the lateral zone of the dorsal telencephalon; Dl-v, ventral part of the lateral zone of the dorsal telencephalon; ICL, internal cellular layer of the olfactory bulb.

The rostral portion of the dorsal part of the lateral zone of the dorsal telencephalon (Dl-dr) was selected as a control region because it is not known to be directly involved in olfactory processing, and its close proximity to the olfactory bulbs allowed quantification on the same brain sections. Dl-dr *cfos*-stained cell densities were similar across all treatment and timing groups (Fig 6; treatment: F = 2.300, P = 0.097; timing: F = 1.473, P = 0.234, timing x treatment: F = 0.007, P = 0.999) indicating that cobalt-treated animals did not have a generalized reduction in brain activation, but rather, the reduction was specific to regions involved in olfactory and lateral line processing.

**Fig 7:**
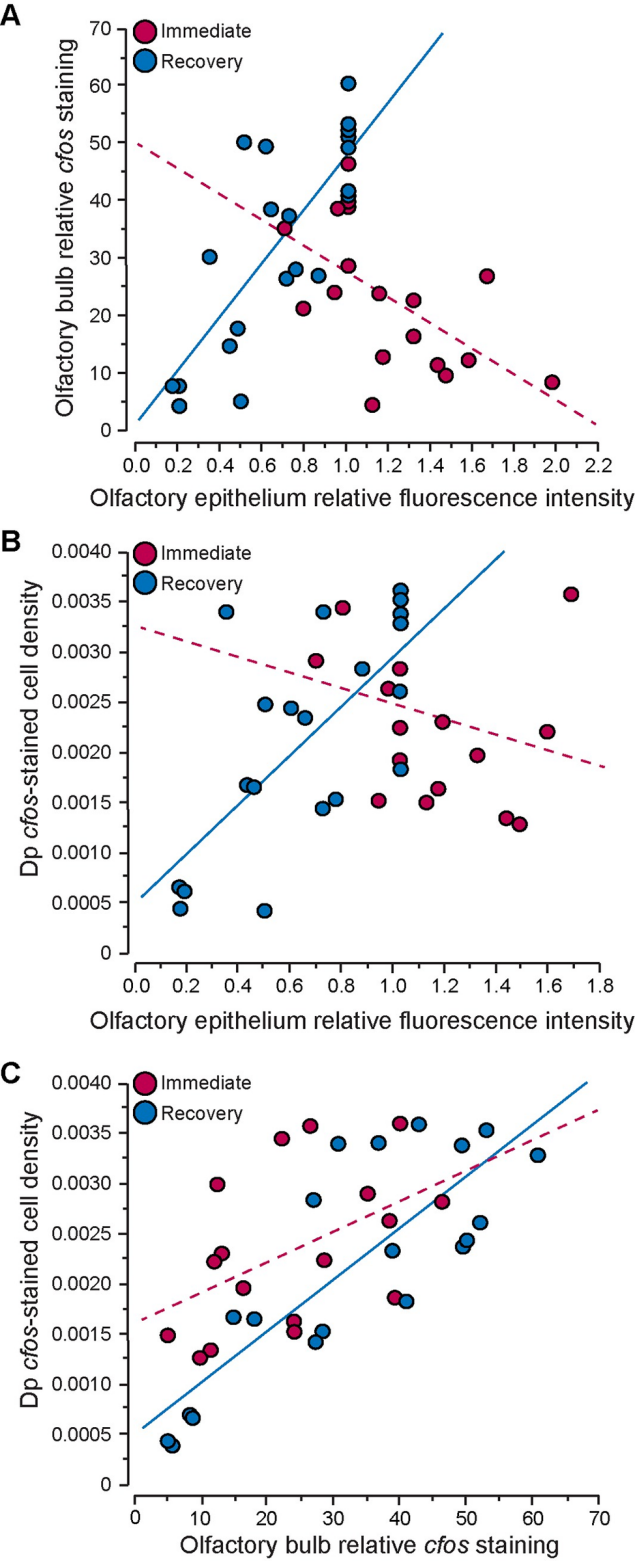
Correlations between fluorescence intensity of the olfactory epithelium and *cfos*-staining in the brain of the cichlid *A*. *burtoni*. (**A-B**) DASPEI-stained fluorescence intensity of the olfactory epithelium is negatively correlated with *cfos*-staining in the olfactory bulb (**A**) and posterior portion of the dorsal telencephalon (Dp, **B**) of fish imaged immediately after treatment (red), but positively correlated in fish allowed to recovery for 18 hours (blue). (**C**) *cfos*-staining in the olfactory bulb and Dp are positively correlated immediately after and at 18-hours after CoCl_2_-treatment. See Table 1 for correlation coefficients and p-values.

To further examine whether fluorescence intensity of the olfactory epithelium is an adequate proxy of activity, we tested for correlations between DASPEI staining of the olfactory epithelium and *cfos* staining in the olfactory bulbs, Dp, and control Dl-dr within the same individual animals (Table 1, Fig 7). Immediately following treatment, DASPEI staining of the OE was negatively correlated with *cfos* staining in the olfactory bulb (R = -0.509, P = 0.026), and *cfos* staining in the olfactory bulbs and Dp was positively correlated (R = 0.490, P = 0.046). In animals that were allowed to recover, DASPEI staining of the OE was positively correlated with *cfos* staining in the olfactory bulb (R = 0.795, P<0.001) and in the Dp (R = 0.684, P<0.001), and *cfos* staining in the olfactory bulb and Dp were positively correlated with each other (R = 0.824, P<0.001). *Cfos*-stained cell densities in the Dl-dr did not correlate with *cfos* staining in other brain regions or with DASPEI fluorescence intensity of the olfactory epithelium (P>0.05 for all).

**Table 1:** Pearson correlation of OE DASPEI-staining and brain *cfos*-staining in *A*. *burtoni*.

Immediate		*cfos* OB	*cfos* Dp	*cfos* Dl	Recovery		*cfos* OB	*cfos* Dp	*cfos* Dl
**FI OE**	R	**-0.509**	0.069	-0.177	**FI OE**	R	**0.795**	**0.684**	0.286
	P	**0.026**	0.793	0.497		P	**<0.001**	**<0.001**	0.208
***cfos* OB**	R		**0.490**	0.081	***cfos* OB**	R		**0.824**	0.083
	P		**0.046**	0.758		P		**<0.001**	0.714
***cfos* Dp**	R			-0.056	***cfos* Dp**	R			0.085
	P			0.827		P			0.723

Bold indicates significance at P<0.05. *Abbreviations*: Dl: rostral portion of the dorsal part of the lateral zone of the dorsal telencephalon; Dp: posterior part of the dorsal telencephalon, FI: fluorescence intensity; OB: olfactory bulb; OE: olfactory epithelium.

## Discussion

We show that treatment with the chemical compound cobalt chloride, which is traditionally used to selectively ablate the mechanosensory lateral line system, also affects olfaction. Peripheral effects were verified with DASPEI staining of the olfactory epithelium in three different freshwater fish species historically used for lateral line research, a cichlid fish *Astatotilapia burtoni*, goldfish, and the Mexican blind cavefish. Although previous research suggested that sub-lethal cobalt doses have no impact on olfaction [57,58], our data show that the commonly used dose of 0.1mM CoCl_2_ for 24 hours had profound impacts on the olfactory system of both goldfish and cavefish. Despite little effect of the low-0.1mM CoCl_2_ treatment (for 3 hours) on DASPEI staining of lateral line neuromasts, it and the high-2mM CoCl_2_ treatment impaired the olfactory system in *A*. *burtoni*. In addition to deleterious effects on the olfactory epithelium of all three species, we found that CoCl_2_-treated *A*. *burtoni* had reduced neural activation (measured via immediate early gene expression) in olfactory processing regions of the brain. Our results demonstrate that in addition to ablating the lateral line system of fishes, CoCl_2_ treatment has significant negative effects on the olfactory system. This discovery warrants some re-evaluation of past research on behavioral functions attributed to the mechanosensory system, particularly those involving contexts where impaired chemoreception is relevant, such as feeding and social interactions.

### Heavy metal toxicity and impacts on chemosensory systems

Exposure to the metal cobalt chloride had negative effects on the olfactory system of goldfish, cavefish, and the cichlid. Heavy metals are known to have toxicity effects on olfaction in fishes (reviewed in [49]). Because dissolved copper is a common environmental contaminant, it is the focus of most research examining the impact of heavy metals on fish behavior and chemosensory functions. For example, fish exposed to copper-contaminated water show altered behavioral states and actively avoided high copper concentrations [54]. In addition, dissolved-copper causes cell death in lateral line neuromasts and cells of the olfactory epithelium [50–53,76]. Copper exposure also reduces the response of the olfactory bulb to amino acids, which are representative of feeding cues [77]. While the impact of copper on chemosensory function has been studied, the impact of cobalt is less clear. Hansen et al (1999) found that Chinook salmon (*Oncorhynchus tshawytscha*) and rainbow trout (*Oncorhynchus mykiss*) avoided water contaminated with sub-lethal concentrations of dissolved cobalt [54], and Janssen showed that high (2mM) CoCl_2_ doses caused altered swimming behavior and increased mucus production in the Mexican blind cavefish [55].

Several studies using cobalt chloride to chemically ablate the lateral line system cite previous research from two studies as evidence that CoCl_2_ treatment does not affect olfaction. First, Yoshii and Kurihara (1983) recorded amino acid-evoked olfactory bulb electrical responses while perfusing various cation-containing solutions over the olfactory epithelium and found that responses to amino acids were unaffected by the presence of 0.1mM Co^2+^ in their perfusion solution in carp (*Cyprinus carpio*), rainbow trout (*O*. *mykiss*), and bullfrog [58]. In addition, Brown (1982) stated that cobalt only inhibited olfactory bulbar electrical activity at concentrations that exceeded its lethal limit [57]. The conclusion of these previous two studies was that sub-lethal doses of CoCl_2_ did not impair olfactory function. Although both of these experiments are commonly referenced as evidence that CoCl_2_ treatment does not impair olfaction, it is important to note that their experimental conditions drastically differed from most studies that use CoCl_2_ to ablate the lateral line system. For example, the simple amino acids tested in these studies are very different from more complex chemicals associated with chemosensory communication. In addition, these two studies only perfused the cobalt solution over the olfactory epithelium for the duration of the experiment (typically <2 hours); whereas, most behavioral studies using CoCl_2_ leave animals in the CoCl_2_ solution for extended periods of time (up to 24 hours). Cobalt, which is noted for its delayed lethal effect [78], could also have a delayed impact on olfaction. For example, the studies by Yoshii and Kurihara (1983) and Brown (1982) only tested the impact of cobalt immediately after treatment, but our results indicate that its impact on olfaction continues even when fish are placed into cobalt-free solutions following a short 3-hour treatment. We hypothesize that this delayed effect is due to cobalt accumulation on the olfactory epithelium and axonal transport to the olfactory bulb or other upstream targets. In contrast to the studies by Brown (1982) and Yoshii and Karihara (1983), Schmachtenberg (2006) found that 2mM cobalt chloride blocked calcium influx in crypt cells (an olfactory receptor neuron type thought to detect pheromones [79]) of the olfactory epithelium in Pacific jack mackerel *Trachurus symmetricus* [22]. Because of experimental differences and species variability, it is important to test the impact of CoCl_2_ treatment on olfaction in a setting that mimics its use in studies on the lateral line system.

### CoCl_2_-treatment impairs olfaction in freshwater fish

DASPEI is primarily used to verify treatment efficacy of aminoglycosides and CoCl_2_ on neuromasts of the lateral line system; however, it also stains many other neural and non-neural cell types [63]. Although an exact uptake mechanism remains unknown, cells must be metabolically active to take in the mitochondrial dye, and metabolic activity is correlated with fluorescence intensity [63]. Meyers et al (2003) found that a similar styryl dye, FM1-43, is rapidly taken up by cells through mechanotransduction and ion channels and more slowly by endocytosis [64], indicating that only alive, functioning cells will stain with FM1-43. In addition to viewing neuromasts with DASPEI staining, we quantified DASPEI-stained fluorescence intensity of the olfactory epithelium. To our knowledge this is the first experiment to use DASPEI to examine olfactory function via staining of the olfactory epithelium. This technique is simple and quick, and can be easily done when examining treatment efficacy on lateral line neuromasts to simultaneously test for and report data on olfactory effects.

In the present study, CoCl_2_-treated *A*. *burtoni* had an initial increase in DASPEI-staining of the olfactory epithelium, which is likely reflective of increased cellular activity. As a calcium channel antagonist, high cobalt concentrations can alter intracellular calcium levels. It is possible that upon this imbalance, altered calcium-dependent mitochondrial activity or increased ion channel activity could result in increased dye-uptake [63]. After prolonged calcium deficits and altered metabolic activity, however, cells begin to die, resulting in little to no DASPEI uptake, which is reflective of the decreased fluorescence intensity in all CoCl_2_-treated goldfish, cavefish, and in *A*. *burtoni* imaged 18-hours post-treatment. Because of the extended treatment for goldfish and cavefish (24-hours vs 3-hours for *A*. *burtoni*), this initial increased cellular activity (i.e. increased fluorescence) had likely already occurred by the end of treatment and was therefore not detected at the immediate sampling point.

Despite the initial increase in DASPEI staining of the olfactory epithelium in *A*. *burtoni*, olfaction was already impaired, as evident by the reduced *cfos* staining in the olfactory bulb of CoCl_2_-treated fish. Neural activation was concentration-dependent such that *A*. *burtoni* treated with low-0.1mM CoCl_2_ had more *cfos* staining in the olfactory bulbs than fish treated with high-2mM CoCl_2_, and both had lower activation than control animals. Interestingly, when examined 18-hours post-treatment, animals treated with low-0.1mM CoCl_2_ had similar *cfos*-staining to the low-calcium animals but both were lower than untreated-control fish. The decreased activation observed in low-calcium fish could be an artifact of the differences in the overnight recovery environment or a result of the low-calcium treatment solution [80,81]. As the low-calcium and untreated-control animals did not differ in any other measure (DASPEI staining intensity or *cfos*-stained cell density in the Dp), it is likely not a result of the calcium-free solution, although more research is still needed to fully understand how calcium-free solutions might impair sensory function. At 18-hours post-treatment, fish treated with high-2mM CoCl_2_ still had reduced activation in the olfactory bulb, further supporting the hypothesis that olfaction does not recover even after 18 hours in a calcium-rich solution (250–500μM). This is significant because this is within the time frame that most behavioral tests on lateral line function are conducted.

Cichlids treated with high-2mM CoCl_2_ had reduced staining in the Dp at 18-hours post-treatment but not immediately following treatment. It is possible for cobalt to accumulate on the olfactory epithelium, be taken into the cells, and then transported to the olfactory bulb [82]. In fact, cobalt was previously used for neuroanatomical tract tracing studies because of its rapid transport down nerves [83]. In addition, studies in rodents showed that intranasal exposure to cobalt-containing solutions results in the presence of cobalt ions in various brain locations, which indicates that cobalt is taken up from the olfactory mucosa and transported into the brain where it can continue to act as a calcium channel antagonist [84]. This is in agreement with our data where we observed decreased neural activity in the Dp only at 18-hours post-treatment. Although we only examined *cfos* staining in the Dp and one other control region, extra-bulbar projections from the olfactory epithelium also project directly to regions of the brain implicated in social behaviors (such as the ventral nucleus of the ventral telencephalon, Vv, and preoptic area) [73,85–87] suggesting that they could also be directly affected by CoCl_2_ treatment. If this is the case, cobalt chloride treatment could have even more widespread effects on neural circuits and social behaviors at multiple levels within the brain.

### Use of CoCl_2_ in behavior studies: social interactions and reproduction

Complex behavioral displays performed during inter- and intra-sexual social interactions can include visual, auditory, olfactory, tactile, and hydrodynamic signals that convey information about the sender’s sex, motivation, reproductive state, and social status, and these behaviors are widely used across fish taxa. Studying the behavioral functions mediated solely by the lateral line system is often problematic because of the difficulty in completely ablating the system, as well as the species-specificity [14,17,19–21,59,88] of many of the available pharmacological/chemical approaches. Cobalt chloride has been commonly used to chemically ablate the mechanosensory lateral line system to examine how impaired reception of water movement signals influences fish behaviors, such as prey detection and social interactions [13,14,19]. However, there is only limited information on the effects of CoCl_2_ on other senses that may be important during multisensory behaviors.

Since the first descriptions of the lateral line system, researchers have proposed that water movement cues could be used for communication during social interactions. Many fish species perform motions that generate water movements as part of their behavioral repertoire [3,60,89–96]. These behaviors produce visual cues in the form of fin and body movements, but also produce water movements that can be detected by the lateral line system of other near-by fish [97]. Recently, researchers have sought to empirically test the role of mechanoreception during social interactions [17,19,20]. A few of these studies used CoCl_2_ to chemically ablate the lateral line system to compare interactions between lateral line-intact and–ablated fish. This methodology, however, poses several potential problems. First, many studies failed to show treatment efficacy on the lateral line system, and instead have relied on previous literature and altered behavior as a verification. Neither of these are adequate to demonstrate reduced mechanoreceptive capabilities, as CoCl_2_ concentrations are species-dependent and also depend on the ionic composition of the solution [88]. CoCl_2_ can also cause toxicity effects leading to altered behavioral states [55], and as we demonstrate here, CoCl_2_ treatment can impair chemosensory function that is likely involved in these social interactions.

The deleterious effect of CoCl_2_ on chemosensory function has important implications for the interpretation of behavioral studies examining the role of mechanoreception, particularly in terms of social interactions. The importance of chemosensory communication during fish social behaviors is well documented, especially in the goldfish *C*. *auratus*. Both male and female goldfish release pheromones that act to prime the opposite sex for reproduction and synchronize spawning [98–101]. Pheromones are typically defined as substances (or mixtures of substances) that individuals release into the environment and when detected by conspecifics, induce adaptive behavioral and physiological responses [65,102]. Many fishes use pheromones as a key communication mode that can influence the behavior and physiology of near-by conspecifics [103–107]. It is well demonstrated that many fish species use contextual urine release to convey information to conspecifics. For example, male tilapia *Oreochromis mossambicus* increase urine release in the presence of gravid, reproductively receptive females, but not when exposed to unreceptive females [103,104,108]. In addition, male swordtails (*Xiphoporus spp*.*)* increase urination frequency in the presence of conspecific females [107], and *A*. *burtoni* dominant males use contextual urine release to signal to gravid females and other territorial males [94]. Similarly, *O*. *mossambicus* males use urinary communication to convey dominance status such that released urinary odorants may modulate aggression from conspecifics [108]. Maruska and Fernald (2012), found that chemical communication varied under different sensory conditions (visual only vs full contact [94]), highlighting the importance of chemical communication during multimodal social interactions. These studies demonstrate the importance of chemosensory signaling during social interactions. Disrupting this key communication channel could have devastating effects on social interactions and even reproductive fitness.

In our previous study, we examined the role of mechanosensory signals in mediating territorial interactions in *A*. *burtoni* [17]. Using CoCl_2_ treatment to ablate the lateral line system, we showed that mechanosensory signals were used for opponent assessment, and that lack of mechanosensory input (decreased assessment abilities) resulted in reduced social interactions. Had this study not verified treatment efficacy and included toxicity controls, the decreased social interactions could have been explained by toxicity effects leading to decreased activity. In addition, comparisons to anosmic controls (ablation of the olfactory epithelium) in this study allowed us to be confident that the observed behavioral changes were due to decreased mechanoreceptive capabilities and not due to decreased chemosensory input.

Of the handful of studies using CoCl_2_ treatment during behavioral studies, only one has controlled for potential unwanted impacts of CoCl_2_ on chemosensory systems [17]. Because of this, the use of CoCl_2_ treatment in studies examining the role of mechanoreception need to be re-evaluated with the understanding that CoCl_2_-treated fish likely have impaired mechanosensory *and* chemosensory systems. An alternative approach used by Satou et al (1994) exposed fish to a vibrating sphere instead of a vibrating conspecific [20]. In these experiments, chemosensory cues were not present, so the role of mechanosensory cues alone can be examined. However, in the context of multimodal social interactions, it is possible that mechanosensory information alone could act differently in a different sensory environment [94, 109]. For the continued use of CoCl_2_ in behavior research, however, all studies should examine for disruptive chemosensory effects and include appropriate controls.

### Use of CoCl_2_ in behavior studies: prey detection and feeding

Outside of reproduction, feeding is one of the most important aspects of a fish’s life. The acquisition of food involves searching for, detecting, capturing, and ingesting food. Depending on the species, prey detection is mediated strongly by visual cues, but other non-visual sensory modalities such as olfaction, gustation, and mechanoreception are likely also involved. Further, the relative importance of each sensory modality may depend on the sensory environment in which the fishes are foraging. Some fish species primarily forage at night, suggesting they rely more on non-visual cues for prey detection. Several studies have investigated the role of the lateral line system in mediating prey detection and capture [12–14]. In one study, researchers sought to understand the role of mechanoreception in night-time feeding strategies [14]. The authors compared the ability of the peacock cichlid, *Aulonocara stuartgranti*, to detect and capture live and dead prey during both light and dark trials. In addition, they used CoCl_2_ treatment to ablate the lateral line system and found that the CoCl_2_-treated fish were unable to detect/capture prey in the dark. While the authors attribute this to the disabled lateral line system, it is also possible that the CoCl_2_-treated fish had impaired chemoreception that decreased their ability to detect prey. The authors later confirmed that *A*. *stuartgranti* respond to artificial flows that mimic the natural hydrodynamic stimuli of prey [61]. After treatment with CoCl_2_, the fish failed to respond to the artificial stimulus, demonstrating that the hydrodynamic cues are used to detect water movements similar to those produced by prey. It is important to point out that fish were conditioned to respond to the stimulus using a food-reward. It is still possible, therefore, that after CoCl_2_ treatment fish had reduced motivation for the food reward or altered activation of neural circuits involved in prey capture. Interestingly, a similar study in *Tramitichromis* sp., which have narrow lateral line canals, found that cobalt-treated and control fish had similar prey capture rates in the light, suggesting that hydrodynamic cues (and potentially chemosensory cues) are less important to this day-time feeder [110]. Acquisition of food in the dark likely involves input from all non-visual sensory systems and to fully understand the relative role of each system in prey detection, more controlled experiments are needed to verify that the CoCl_2_ treatments previously used had no impact on chemosensory systems.

### Recommendations for future use of CoCl_2_ in lateral line research

Cobalt chloride treatment has been a common tool for chemical ablation of the lateral line system for the past 30 years. However, due to behavioral and toxicity effects shown at high concentrations for prolonged periods in some species [55], and the now demonstrated comorbid impact on chemosensory systems, the continued use of CoCl_2_ in behavioral studies of the lateral line system should be performed with caution. Moving forward, it would be advantageous to no longer use CoCl_2_ in any lateral line research and instead switch to other methods. Since our results show that CoCl_2_ treatment also impairs olfaction (and likely gustation, although not studied here), we recommend that CoCl_2_ (and other heavy metals) be used for chemical ablation of the lateral line system only as a last resort. Aminoglycoside antibiotics offer a nice alternative to CoCl_2_ and have been successfully used in many recent lateral line studies. One limitation of aminoglycosides is the high variability of effectiveness across different species [88], and that aminoglycoside efficacy is dependent on the ionic composition of the solution. Like cobalt, antibiotics and calcium appear to have antagonizing effects on each other [111–113]. Gentamicin and streptomycin used in high conductivity water are less effective than when used in RO water [17,88], which precludes their use for many marine fishes. Further, one study noted a high mortality rate associated with antibiotic treatment [114], leading some to question their results. An alternative to chemical and pharmacological ablation is the use of physical ablation techniques, such as transections of the lateral line nerves to remove input to central lateral line processing regions [18,59]. While exposure and transection of the posterior lateral line nerves is quick and easy, the anterior lateral line nerves (ALLn) often travel in close proximity to other cranial nerves and the brain, making transection of all ALLn branches more difficult. Scrapping, plucking, ‘peeling off’ superficial neuromasts after glue application, and burning off neuromasts with liquid nitrogen are other physical ablation methods that have been previously used [88,115,116]. As with chemical ablation techniques, however, appropriate controls are still needed to ensure treatment efficacy and control for handling and treatment stress.

When the use of CoCl_2_ is unavoidable, researchers should minimally verify treatment efficacy using a vital dye, such as DASPEI or FM1-43. In addition to revealing treatment effectiveness on lateral line neuromasts, vital dye staining will reveal potential impacts on the olfactory epithelium, making it relatively easy for researchers to verify effects on lateral line and olfaction in the same animals. Finally, researchers using CoCl_2_ should include appropriate controls to demonstrate that any behavioral measures quantified are not due to toxicity or impacts on other sensory systems. For example, our previous work in *A*. *burtoni* included anosmic (olfactory epithelium ablated) controls and allowed us to be confident that behavioral changes were not due to lack of chemoreception [17]. Our results shown here now suggest that any study using CoCl_2_ treatment must control for CoCl_2_ effects on chemosensory systems. Understanding the role of mechanosensory signals during social interactions can provide important and relevant information on the relative importance of individual sensory channels during multimodal behaviors. With more than 30,000 species of fishes, and most species possessing both lateral line and chemosensory systems, it is important to distinguish between these two sensory systems in future research if we hope to fully understand the role of the mechanosensory lateral line in mediating behaviors.

## Conclusion

Results of this study demonstrate that a common treatment used to ablate the mechanosensory lateral line system, CoCl_2_, also impairs the olfactory sense in three different species of freshwater fishes. Many fishes use chemosensory signaling during social interactions, and detection of chemical compounds is essential for predator avoidance and prey detection. The lateral line system is also implicated in these behaviors, however, most previous studies failed to examine potential effects of cobalt chloride on chemosensory systems. Fishes use multisensory signaling during social interactions and this information is integrated in the brain to produce context-appropriate behaviors. The role of each sensory channel in providing unique or redundant information to the receiver is currently the interest of many research programs. It is important, therefore, to verify that any treatment intended to remove one sensory channel does not disrupt use of other sensory modalities.

## Supporting Information

S1 Dataset Excel file containing data used for analysis. Dataset includes all fish data, DASPEI-fluorescence data, and *cfos* quantification data for each species. Data from *A*. *burtoni*, goldfish, and cavefish are on individual sheets, and all abbreviations are defined within each sheet.
(XLSX)
